# Roles of myeloperoxidase and the AMPK/PI3K/AKT/eNOS pathway in osimertinib-induced cardiotoxicity: multilevel evidence from disequilibrium analysis, network pharmacology, mendelian randomization, and animal experiments

**DOI:** 10.3389/fphar.2026.1776465

**Published:** 2026-07-01

**Authors:** Jing Shi, Xinya Liu, Jian Yu, Li Wu, Yun Jiang, Mengjiao Gao, Yuanming Zhang

**Affiliations:** 1 Department of Oncology Cardiology, Affiliated Cancer Hospital of Xinjiang Medical University, Urumqi, China; 2 Xinjiang Medical University, Urumqi, China; 3 The Fifth Affiliated Hospital of Xinjiang Medical University, Urumqi, China

**Keywords:** animal experiments, disproportionality analysis, epidermal growth factor receptor, FAERS database, mendelian randomization, network pharmacology, osimertinib

## Abstract

**Introduction:**

Osimertinib, a third-generation Epidermal Growth Factor Receptor-Tyrosine Kinase Inhibitor (EGFR-TKI), is central to first-line therapy for EGFR-mutant Non-Small Cell Lung Cancer (NSCLC), showing improved outcomes; however, its cardiotoxicity raises critical concerns. The incidence, risk factors, and management of this condition remain poorly understood and require further mechanistic exploration. This study aimed to investigate the risk signals, mechanisms, and causal associations of osimertinib-induced cardiotoxicity using multilevel analyses, focusing on myeloperoxidase (MPO) and the AMPK/PI3K/AKT/eNOS pathway.

**Methods:**

The study methods included: (1) disproportionality analyses of FAERS and VigiBase (Q1 2016-Q4 2024), (2) network pharmacology for targets/pathways, (3) Mendelian randomization for causal validation, and (4) animal experiments to test the mechanisms. The exposures were osimertinib in real-world data; in animals, the exposures were osimertinib (8.33 mg/kg, i. p.) alone or with inhibitors (ABAH, Compound C, LY294002, L-NAME). The main outcomes and measures were (1) Real-world: CAE incidence/spectrum/risk signals (ROR, PRR, IC; e.g., heart failure, arrhythmias) (2) Molecular: Core targets/pathways (e.g., PI3K/AKT). via network pharmacology and genetically validated targets via Mendelian randomization. (3) *In vivo*: Cardiac function (LVEF, LVFS), ECG (QT), myocardial damage, apoptosis, oxidative stress (MDA, SOD), and inflammation (IL-1β, IL-6, TNF-α).

**Results:**

Disproportionality analyses showed high signals for cardiac dysfunction (ROR = 16.10, FAERS), cardiotoxicity (ROR = 8.43, FAERS), and long QT syndrome (ROR = 15.85, VigiBase). Network pharmacology identified 111 overlapping targets with core targets in the PI3K/AKT pathway. Mendelian randomization validated nine targets related to PI3K/AKT with causal ties to arrhythmias/heart failure. In animal experiments, osimertinib upregulated MPO, inhibited AMPK/PI3K/AKT/eNOS, and induced cardiac dysfunction, damage, apoptosis, oxidative stress, and inflammation. MPO inhibition attenuated these effects, and co-administration with pathway inhibitors reversed the protective effect.

**Discussion:**

Osimertinib induces cardiotoxicity via MPO upregulation, which inhibits the AMPK/PI3K/AKT/eNOS pathway. MPO and related pathways may serve as biomarkers or therapeutic targets, supporting risk monitoring and safety management in osimertinib use.

## 1 Introduction

As a third-generation EGFR tyrosine kinase inhibitor (EGFR-TKI), osimertinib has an important application as the first-line therapy for EGFR-mutant non-small cell lung cancer (NSCLC) and markedly improves patient outcomes (Xia et al., 2024; Mirza et al., 2024). However, its potential cardiotoxicity represents a critical concern in clinical practice. Although important clinical trials have hinted at related risks, core issues such as the real-world incidence of osimertinib-induced cardiotoxicity, specific risk factors, and standardized management strategies have not been clarified.

A large real-world retrospective study reported that the incidence of osimertinib-related cardiotoxicity, including reduced left ventricular ejection fraction, symptomatic heart failure, new-onset arrhythmias, and cardiac death, was 4.7% (Bak et al., 2025). Advanced age, history of heart failure, atrial fibrillation, and reduced baseline left ventricular strain were identified as independent risk factors. Although 82.4% of the cardiac adverse events (CAEs) were reversible, such events were significantly associated with increased mortality (HR = 1.49). Despite enhanced credibility through a thorough case review, this study had limitations, including a relatively short median drug exposure duration (12.4 months) and a low proportion of first-line treatment patients (7%), which diverges from real-world clinical practice. Moreover, the definition of cancer therapy-related cardiotoxicity remains unstandardized, and issues such as the combined risks of osimertinib with chemotherapy, underlying toxicity mechanisms, and management strategies for high-risk populations require further exploration, underscoring the necessity and urgency of in-depth research.

## 2 Methods

This study adopted a multilayered analytical strategy to systematically investigate the potential risk signals, molecular mechanisms, and causal associations of osimertinib-induced cardiotoxicity. We performed disproportionality analyses—via Food and Drug Administration (FDA) FAERS/WHO VigiBase—with ROR/IC models to quantify osimertinib-cardiotoxicity associations and capture risk signals (Shi et al., 2025; Shi et al., 2024a; Shi et al., 2024b). Network pharmacology, integrating drug structure, targets, and cardiotoxicity-related genes, was performed to construct “drug-target-toxicity” networks, identifying core targets/pathways (Nogales et al., 2022). Summary data Mendelian randomization (using genetic variants as instrumental variables) was performed to validate causality, avoiding confounding or reverse causation (Zhu et al., 2016; Wu et al., 2018; Qi et al., 2018).

These multidimensional analyses, progressing from clinical associations and molecular mechanisms to causal validation, lay the foundation for a scientific understanding of osimertinib-induced cardiotoxicity. Building on this, this study further conducted *in vivo* validation through animal experiments, aiming to more directly clarify the toxic effects and mechanisms of osimertinib, thus providing more comprehensive evidence for clinical risk prevention and control.

**FIGURE 1:**
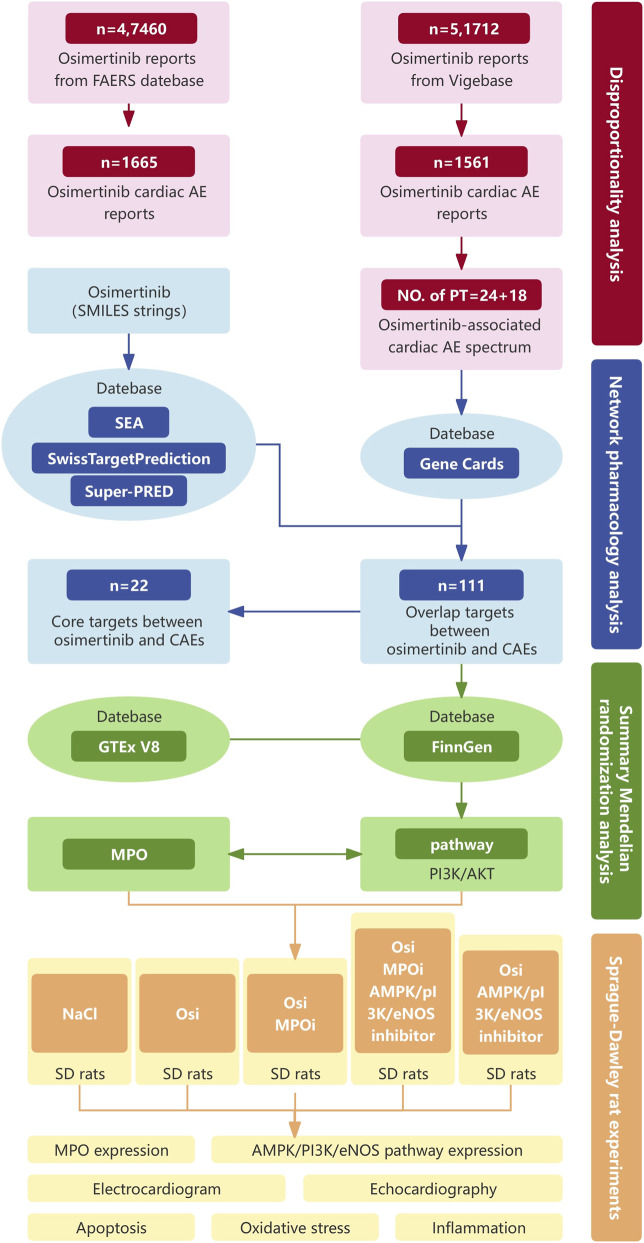
Flowchart of Osimertinib-related AEs investigation.

The methodological overview is illustrated in Figure 1.

### 2.1 Disproportionality analysis

Data were sourced from the U.S. Food and Drug Administration (FDA) Adverse Event Reporting System (FAERS) and the World Health Organization Global Individual Case Safety Report Database (VigiBase) covering reports from the first quarter of 2016 to the fourth quarter of 2024. The inclusion criteria were as follows: the latest patient reports containing complete age information and adverse event terms were standardized using the Medical Dictionary for Regulatory Activities (MedDRA version 27.1).

Using “Osimertinib” as the keyword, information on event classifications such as System Organ Classes (SOCs) and Preferred Terms (PTs) was extracted, along with clinical characteristics including patient sex, age, time to onset (interval from drug administration to event occurrence), and mortality. Data deduplication followed the FDA guidelines: reports with the latest FDA receipt date were retained via CASEID; for records with the same CASEID and receipt date, the one with the highest PRIMARYID was selected; and reports from database deletion files starting from the first quarter of 2019 were excluded. Statistical analyses were performed using SAS 9.4. Based on a 2 × 2 contingency table (Table 1), the observed and expected reporting rates of the drug-event pairs were compared. Three methods were combined: reporting odds ratio (ROR, with ROR ≥2 and the lower bound of the 95% confidence interval [CI] > 1), proportional reporting ratio (PRR, with PRR ≥2 and the lower bound of the 95% CI > 1), and Bayesian confidence propagation neural network (BCPNN, with information component IC02 ≥ 0. A safety signal was determined only when all the three methods met the positive criteria (Table 2).

**TABLE 1:** Two-by-two contingency table for disproportionality analysis.

Item	Target adverse events reported	Other adverse events reported	Total
Target drugs	a	b	a + b
Other drugs	c	d	c + d
Total	a + c	b + d	a + b + c + d

**TABLE 2:** Principles of disproportionate measurement and criteria for signal detection.

Algorithms	Equation	Criteria
ROR	$\text{ROP}=\frac{\left(a/c\right)}{\left(b/d\right)}=\frac{ad}{bc}$	Lower limit of 95% CI > 1, N ≥ 3
	95% CI = eln (ROR) ± 1.96 (1/a+1/b+1/c+1/d)^0.5^	
PRR	$\text{PRR}=\frac{a/\left(a+b\right)}{c/\left(c+d\right)}$	Lower limit of 95% CI > 1, N ≥ 3
	$x^{2}=\frac{{\left(ad-bc\right)}^{2}\left(a+b+c+d\right)}{\left(a+b\right)\left(a+c\right)\left(c+d\right)\left(b+d\right)}$	
BCPNN	$95\%CI=e^{In\left(PRR\right)\pm 1.96}\sqrt{\frac{1}{a}-\frac{1}{a+b}+\frac{1}{c}-\frac{1}{c+d}}$	
	$\text{IC}=log^{2}=\frac{p\left(x,y\right)}{p\left(x\right)p\left(y\right)}={log}^{2}\frac{a\left(a+b+c+d\right)}{\left(a+b\right)\left(a+c\right)}$	IC025 > 0, N ≥ 3
	95% CI = E (IC) ± 2V(IC)^0.5^	

95% CI: 95% confidence interval.

ROR: reporting odds ratio.

PRR: proportional reporting ratio.

IC: information component.

### 2.2 Network pharmacology analysis

The chemical information of osimertinib (including its molecular formula, SMILES string, and 2D and 3D structures) was obtained from the Drug Bank database (version 5.1.10). Potential targets were predicted through three platforms: Similarity Ensemble Approach (SEA) database (ligand similarity algorithm, Max TC ≥ 0.3, referencing the target prediction threshold for similar targeted drugs), Super-PRED database (machine learning model, prediction probability ≥50%), and SwissTargetPrediction database (integrated molecular descriptors, probability ≥0.1). Relatively permissive screening thresholds were applied in the initial prediction of Osimertinib targets to minimize false negatives and maximize target coverage. Potential false positives were subsequently eliminated through rigorous molecular docking and network topological analysis, ensuring the specificity of the final identified targets. The union of the targets from these three platforms was used to construct the potential target profile of Osimertinib.

Disease names that were positive in all three methods of the disproportionality analysis were converted into Medical Subject Headings (MeSH) after clinical and literature semantic verification. Thereafter, they were used to search for associated targets in the GeneCards database (version 5.11), retaining those with a relevance score ≥10, based on the conventional threshold for screening cardiac disease targets. A Venn diagram was drawn using Venny 2.1.0 to screen the intersection of potential targets of osimertinib and cardiac disease targets (candidate targets). The STRING database (V12.0, with species set to human and confidence >0.7) was used to construct a protein-protein interaction (PPI) network, which was visualized using Cytoscape 3.10.3. Core targets were screened according to the following criteria: betweenness centrality > median, closeness centrality > median, average shortest path length > median, and degree value > two times the median. DAVID 6.8 was used for Gene Ontology (GO) functional enrichment and Kyoto Encyclopedia of Genes and Genomes (KEGG) pathway enrichment analyses of core targets (with P < 0.05, and False Discovery Rate [FDR] < 0.25). Results were visualized using a bioinformatics platform (https://www.bioinformatics.com.cn/).

### 2.3 Summary data-based mendelian randomization analysis

The disease names identified in the preceding analysis were converted into MeSH terms using the official website of the National Library of Medicine to standardize the retrieval process. Genome-Wide Association Study (GWAS) data corresponding to these diseases were obtained from the FinnGen database (R9), and expression Quantitative Trait Loci (eQTL) data were retrieved from the Genotype-Tissue Expression (GTEx) project (v8).

For instrumental variable selection, cis-eQTLs within each gene region were chosen as genetic instruments, and only variants with P < 1 × 10^−5^ were included to ensure sufficient instrument strength and minimize weak instrument bias.** Quality control criteria were applied as follows: minor allele frequency >0.01, Hardy–Weinberg equilibrium P > 1 × 10^−6^, and linkage disequilibrium *r* ^2^ < 0.01. Analyses were conducted using the SMR tool (version smr-1.3.1-win).

Horizontal pleiotropy was formally evaluated using the HEIDI test, with p_HEIDI >0.05 indicating no evidence of pleiotropic bias and supporting the robustness of causal inference. The threshold for significant association was set at p_SMR <0.05 and *p*_HEIDI >0.05, consistent with conventional standards in genetic epidemiological studies.

### 2.4 Animal experiments

#### 2.4.1 Animals

A total of 100 SPF-grade male SD rats aged 6 weeks (body weight 220 ± 20 g) were purchased from Sibeifu (Suzhou) Biotechnology Co., Ltd. (license number: B202410140040). All experiments were approved by the Laboratory Animal Welfare and Ethics Committee of the Zhejiang Provincial Laboratory Animal Center and conducted in accordance with the 3R principles (Replacement, Reduction, Refinement). During the experiment, euthanasia was performed under deep anesthesia to ensure that the animals were fully unconscious. The feeding environment was set as follows: room temperature 22 °C ± 2 °C, relative humidity 60%–80%, 12 h light-dark cycle, with free access to food and water.

#### 2.4.2 Experimental design

All SPF-grade male Sprague–Dawley (SD) rats were used in the present study due to their stable genetic background, uniform physiological traits, and widespread application in cardiovascular and cardiotoxicity research, which guarantees good reproducibility and comparability with previous studies. After 1 week of adaptive feeding under standard conditions, the rats were randomly divided into a control group, a model group, and multiple intervention groups according to the experimental protocol (Table 3).

**TABLE 3:** Animal number and administration dosage of each experimental group.

Group name	Number	Administration dosage
Control group	10	Normal saline, volume consistent with osimertinib in the model group
Model group	10	Osimertinib 8.33 mg/kg
Model + MPO inhibitor group	9	Osimertinib 8.33 mg/kg + ABAH 40 mg/kg
Model + AMPK inhibitor group	10	Osimertinib 8.33 mg/kg + compound C 0.2 mg/kg
Model + PI3K inhibitor group	10	Osimertinib 8.33 mg/kg + LY294002 0.3 mg/kg
Model + eNOS inhibitor group	9	Osimertinib 8.33 mg/kg + L-NAME 30 mg/kg
Model + MPO inhibitor + AMPK inhibitor group	9	Osimertinib 8.33 mg/kg + ABAH 40 mg/kg + compound C 0.2 mg/kg
Model + MPO inhibitor + PI3K inhibitor group	9	Osimertinib 8.33 mg/kg + ABAH 40 mg/kg + LY294002 0.3 mg/kg
Model + MPO inhibitor + eNOS inhibitor group	9	Osimertinib 8.33 mg/kg + ABAH 40 mg/kg + L-NAME 30 mg/kg

The model group and all intervention groups received intraperitoneal injection of osimertinib at a dose of 8.33 mg/kg, which was converted from clinical human exposure levels based on body surface area conversion, thus ensuring the clinical relevance of the animal model. Osimertinib was administered once weekly on the 7th day for 6 consecutive weeks to establish a chronic cardiotoxicity model consistent with the clinical exposure course of targeted anti-tumor therapy. The control group was given an equal volume of normal saline via the same route and schedule.

After successful establishment of the cardiotoxicity model, each intervention group received corresponding targeted inhibitors for 7 consecutive days. Specifically, the MPO inhibitor group was administered 40 mg/kg ABAH by intragastric gavage, the AMPK inhibitor group received 0.2 mg/kg Compound C via intraperitoneal injection, the PI3K inhibitor group was given 0.3 mg/kg LY294002 by intraperitoneal injection, and the eNOS inhibitor group received 30 mg/kg L-NAME via intraperitoneal injection. In addition, three combined intervention groups were established by co-administering the MPO inhibitor ABAH with each of the three pathway inhibitors (Compound C, LY294002, and L-NAME) at the aforementioned doses, in order to further explore the crosstalk and regulatory relationship among MPO and the AMPK/PI3K/Akt/eNOS signaling pathway. All inhibitors were selected based on their high specificity, clear pharmacological mechanisms, and widely recognized safe and effective doses in cardio-oncology studies.

#### 2.4.3 Cardiac ultrasound and electrocardiography

At 24 h after the last administration, the rats were anesthetized with isoflurane via gas. A V6 small animal ultrasound system was used to measure the left ventricular ejection fraction (LVEF), left ventricular fractional shortening (LVFS), left ventricular internal dimension in systole (LVIDs), and left ventricular internal dimension in diastole (LVIDd). A Madlab4c-5H bioinformatics medical information processing system was used to detect electrocardiograms (ECG), and parameters such as heart rate and QT interval were recorded.

#### 2.4.4 Sample collection

After the ultrasound examination, the rats were sacrificed via cardiac exsanguination under deep anesthesia (sodium pentobarbital 50 mg/kg). (1) Serum samples: abdominal aortic blood was centrifuged at 3,000°r/min for 15 min to separate the serum, which was stored at −80 °C. (2) Cardiac tissue: Left ventricular tissue was taken, part of which was fixed with 4% paraformaldehyde (embedded in paraffin after 24 h), and part was snap-frozen in liquid nitrogen and stored at −80 °C for detection analysis such as flow cytometry and Western blot.

#### 2.4.5 Statistical analysis

SPSS 19.0 and GraphPad Prism 8.2 were used for statistical analysis. Measurement data conforming to normal distribution were expressed as mean ± standard deviation (x̅±s). One-way analysis of variance (ANOVA) was used for comparisons among multiple groups, and the LSD-t test (for homogeneous variance) or Dunnett’s T3 test (for heterogeneous variance) was used for pairwise comparisons. Data with a non-normal distribution were expressed as median (25th percentile, 75th percentile) [M (P25, P75)]. The Kruskal–Wallis H test was used for comparisons among multiple groups, and the Bonferroni correction was used for pairwise comparisons. The test level α = 0.05.

## 3 Results

### 3.1 Basic characteristics of osimertinib-related AEs and CAEs in FAERS and VigiBase databases

**FIGURE 2:**
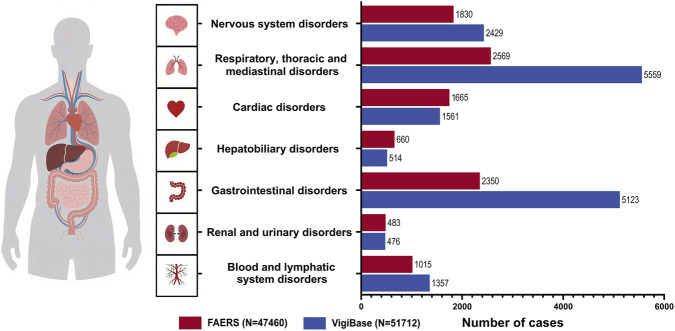
Case numbers of AEs for multiple SOCs in the FAERS and VigiBase Databases.

Retrieval and analysis of the FAERS and VigiBase databases showed that the cumulative number of osimertinib-related AE reports from 2016 to 2024 was 47,460 and 51,712, respectively, among which CAEs accounted for 1,665 and 1,561 cases, respectively. Under the MedDRA SOC framework, the number of CAEs ranked fourth, followed by AEs of the respiratory, digestive, and nervous systems (Figure 2).

**FIGURE 3:**
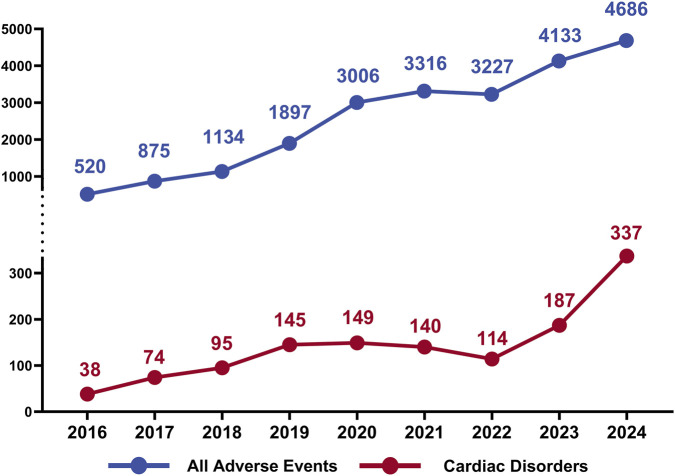
Annual counts of Osimertinib-related AEs in FAERS All vs. Cardiac Disorders.

Time-trend analysis indicated that osimertinib-related AEs have generally shown an annual increasing trend since 2016, with a significant increase over the past 3 years. Notably, the upward trend in CAEs over the past 3 years exceeded the growth level of the overall AEs (Figure 3).

In addition, the analysis of demographic characteristics for osimertinib-related adverse events (AEs) (Table 4) revealed the following: among reports in the FDA FAERS database, 52.56% were female and 33.95% were aged ≥65 years; in the WHO VigiBase database, 59.68% were female and 40.15% were aged ≥65 years. Notably, 48.19% of AE reports in the FAERS database lacked explicit age information; therefore, only reports with complete and documented age data were included in age-stratified analyses in this study. Among cardiac adverse events (CAEs), the proportion of patients aged ≥65 years reached 48.28%, which was higher than that in overall AEs. The hospitalization rate of CAEs was 48.2%, markedly higher than the 17.98% for all AEs. The median time from drug initiation to the onset of CAEs was 45 days, shorter than the 59 days for all AEs.

**TABLE 4:** Characteristics of reports on osimertinib-associated AEs sourced from the FAERS database and VigiBase.

Clinical characteristics	FAERS		VigiBase
​	All AEs	CAEs	All AEs
Gender			
Female (%)	11,990 (52.56)	738 (57.66)	16,005 (59.68)
Male (%)	6,403 (28.07)	354 (27.66)	8,098 (30.2)
Not specified (%)	4,420 (19.37)	188 (14.69)	2,716 (10.13)
Age			
<18 (%)	9 (0.04)	0 (0.00)	22 (0.08)
18–44 (%)	381 (1.67)	11 (0.86)	489 (1.82)
45–64 (%)	3,684 (16.15)	168 (13.13)	5,070 (18.9)
≥65 (%)	7,746 (33.95)	618 (48.28)	10,767 (40.15)
Not specified (%)	10,993 (48.19)	483 (37.73)	10,471 (39.04)
Region			
North America (%)	5,715 (25.05)	492 (38.44)	11,852 (44.19)
Asia (%)	4,090 (17.93)	550 (42.97)	10,350 (38.59)
Europe (%)	1,249 (5.47)	196 (15.31)	4,161 (15.52)
Oceania (%)	–	​	203 (0.89)
Australia (%)	–	16 (1.25)	387 (1.44)
South America (%)	–	23 (1.80)	155 (0.68)
Africa (%)	23 (0.10)	3 (0.23)	69 (0.26)
Not specified (%)	11,378 (49.88)	–	–
Outcome			
Life-threatening (%)	641 (2.81)	166 (12.97)	–
Hospitalization (%)	4,102 (17.98)	617 (48.20)	–
Disability (%)	248 (1.09)	33 (2.58)	–
Death (%)	11,133 (48.80)	294 (22.97)	–
Congenital anomaly (%)	9 (0.04)	2 (0.16)	–
Required intervention to prevent permanent impairment/damage (%)	10 (0.04)	2 (0.16)	–
Other (%)	9,437 (41.37)	755 (58.98)	–
Onset time of AEs (days)			
0–30 days (%)	1889 (8.28)	174 (13.59)	–
31–60 days (%)	704 (3.09)	58 (4.53)	–
61–90 days (%)	387 (1.70)	34 (2.66)	–
91–120 days (%)	293 (1.28)	25 (1.95)	–
121–150 days (%)	204 (0.89)	12 (0.94)	–
151–180 days (%)	172 (0.75)	22 (1.72)	–
181–360 days (%)	659 (2.89)	44 (3.44)	–
>360 days (%)	1,108 (4.86)	55 (4.30)	–
Mean (SD)	60.52 (16.73)	158.05 (274.47)	–
Median (Q1,Q3)	59.00 (49.00.68.00)	45.50 (15.00,172.50)	–
Min,Max	22.90,180.00	0.00,2049.00	–

### 3.2 Cardiac disease spectrum and mortality obtained from disproportionality analysis of osimertinib AEs

After conducting disproportionality analysis on the FAERS and VigiBase databases, CAEs (Preferred Terms) that were positive in the verification by the three methods and had a case count of ≥3 were obtained (Table 5). The top five diseases by signal strength (ROR) in the two databases were as follows: FAERS: cardiac dysfunction (n = 40, ROR = 16.10), cardiotoxicity (n = 54, ROR = 8.43), acute cardiac failure (n = 32, ROR = 6.46), long QT syndrome (n = 17, ROR = 9.15), toxic cardiomyopathy (n = 3, ROR = 14.57); VigiBase: cardiotoxicity (n = 33, ROR = 6.88), cardiac dysfunction (n = 28, ROR = 12.90), long QT syndrome (n = 22, ROR = 15.85), heart failure with reduced ejection fraction (n = 18, ROR = 47.76), stress cardiomyopathy (n = 18, ROR = 6.10).

**TABLE 5:** Detected significant safety signals of CAEs at the preferred term level from the FAERS and VigiBase databases.

Preferred term	N	ROR (95% CI)	PRR (95% CI)	IC(IC025)
FAERS				
Cardiac failure	185	2.99 (2.59,3.46)	2.99(2.59,3.45)	1.58(1.35)
Atrial fibrillation	144	1.91 (1.62,2.25)	1.91 (1.62,2.25)	0.93 (0.68)
Cardiac disorder	138	1.87 (1.58,2.21)	1.87 (1.58,2.21)	0.90 (0.65)
Pericardial effusion	82	4.73 (3.80,5.87)	4.72 (3.80,5.86)	2.23 (1.85)
Cardiomyopathy	66	5.69 (4.47,7.25)	5.68 (4.46,7.23)	2.50 (2.05)
Cardiotoxicity	54	8.43 (6.45,11.02)	8.42 (6.45,11.01)	3.07 (2.49)
Cardiac dysfunction	40	16.10 (11.79,22.00)	16.09 (11.78,21.98)	3.99 (3.09)
Cardiac failure acute	32	6.46 (4.56,9.15)	6.46 (4.56,9.14)	2.68 (1.96)
Left ventricular dysfunction	23	4.54 (3.02,6.84)	4.54 (3.02,6.84)	2.18 (1.39)
Myocarditis	22	2.80 (1.85,4.26)	2.80 (1.84,4.26)	1.48 (0.77)
Cardiac tamponade	21	5.69 (3.71,8.74)	5.69 (3.70,8.73)	2.50 (1.61)
Stress cardiomyopathy	19	4.51 (2.87,7.07)	4.51 (2.87,7.07)	2.17 (1.29)
Long QT syndrome	17	9.15 (5.68,14.75)	9.15 (5.68,14.75)	3.18 (1.97)
Cardiomegaly	17	1.66 (1.03,2.68)	1.66 (1.03,2.68)	0.73 (0.00)
Torsade de pointes	16	2.64 (1.62,4.31)	2.64 (1.62,4.31)	1.40 (0.56)
Supraventricular tachycardia	14	1.87 (1.10,3.15)	1.87 (1.10,3.15)	0.90 (0.07)
Left ventricular failure	12	4.42 (2.51,7.79)	4.42 (2.51,7.79)	2.14 (1.00)
Cardiac failure chronic	11	3.42 (1.89,6.19)	3.42 (1.89,6.19)	1.77 (0.67)
Ventricular dysfunction	10	5.23 (2.81,9.74)	5.23 (2.81,9.73)	2.38 (1.04)
Myocardial injury	10	3.82 (2.05,7.10)	3.82 (2.05,7.10)	1.93 (0.73)
Ventricular hypokinesia	9	3.53 (1.83,6.79)	3.53 (1.83,6.78)	1.82 (0.58)
Dilated cardiomyopathy	9	2.21 (1.15,4.25)	2.21 (1.15,4.24)	1.14 (0.06)
Cardiac discomfort	5	3.25 (1.35,7.83)	3.25 (1.35,7.83)	1.70 (0.06)
Toxic cardiomyopathy	3	14.57 (4.67,45.49)	14.57 (4.67,45.49)	3.85 (0.27)
VigiBase				
Cardiac failure	293	5.56 (4.95,6.23)	5.51 (4.91,6.17)	2.46 (2.27)
Atrial fibrillation	122	1.69 (1.41,2.02)	1.69 (1.41,2.01)	0.75 (0.48)
Cardiac disorder	119	1.94 (1.62,2.33)	1.94 (1.62,2.32)	0.96 (0.68)
Cardiac failure congestive	92	1.68 (1.37,2.06)	1.68 (1.37,2.06)	0.75 (0.44)
Cardiomyopathy	69	5.32 (4.20,6.74)	5.31 (4.19,6.72)	2.40 (1.97)
Pericardial effusion	64	3.76 (2.94,4.81)	3.76 (2.94,4.80)	1.91 (1.49)
Cardiotoxicity	33	6.88 (4.89,9.69)	6.87 (4.88,9.67)	2.78 (2.05)
Cardiac dysfunction	28	12.90 (8.89,18.71)	12.88 (8.88,18.68)	3.68 (2.65)
Cardiac failure acute	23	5.81 (3.86,8.75)	5.81 (3.86,8.74)	2.53 (1.68)
Long QT syndrome	22	15.85 (10.41,24.12)	15.84 (10.41,24.09)	3.97 (2.65)
Left ventricular dysfunction	21	4.82 (3.14,7.40)	4.82 (3.14,7.39)	2.27 (1.42)
Heart failure with reduced ejection fraction	18	47.76 (29.90,76.30)	47.73 (29.89,76.22)	5.54 (3.10)
Stress cardiomyopathy	18	6.10 (3.84,9.69)	6.10 (3.84,9.68)	2.60 (1.60)
Cardiac tamponade	16	5.67 (3.47,9.26)	5.67 (3.47,9.26)	2.50 (1.45)
Dilated cardiomyopathy	13	3.87 (2.24,6.66)	3.87 (2.24,6.66)	1.95 (0.91)
Torsade de pointes	13	2.08 (1.21,3.59)	2.08 (1.21,3.58)	1.06 (0.18)
Cardiac failure chronic	10	4.29 (2.31,7.98)	4.29 (2.31,7.98)	2.10 (0.85)
Ventricular dysfunction	8	5.49 (2.74,10.99)	5.49 (2.74,10.98)	2.45 (0.91)

Red highlighting denotes the AEs with the highest signal strength (ROR, PRR, IC values) in their respective databases.

Regarding the number of occurrences, the top five diseases reported in the FAERS database were cardiac failure (n = 185), atrial fibrillation (n = 144), cardiac disorder (n = 138), pericardial effusion (n = 82), cardiomyopathy (n = 66); in the VigiBase database, they were cardiac failure (n = 293), atrial fibrillation (n = 122), cardiac disorder (n = 119), congestive cardiac failure (n = 92), cardiomyopathy (n = 69).

**FIGURE 4:**
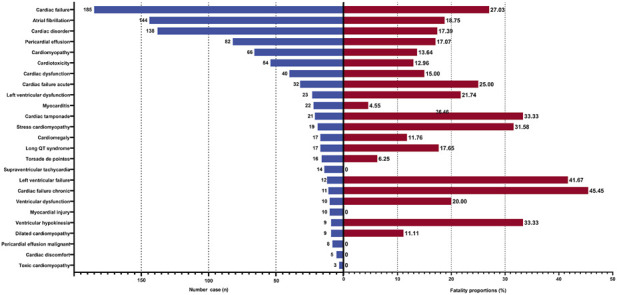
Report numbers and mortality proportion of different CAEs (PT level) associated with Osimertinib in FAERS.

Regarding the number of deaths and the proportions of the above AEs in the FAERS database (Figure 4), cardiac failure had the highest number of occurrences and a relatively high mortality rate (27.03%); chronic cardiac failure had the highest mortality rate, with five deaths out of 11 reported cases (45.45%). Except for seven PTs with a death proportion <10%, the mortality rate of most AEs was 11.11%–45.45%. In the VigiBase database, data on hospitalization, mortality, and clinical outcomes were largely incomplete for cardiac adverse events (CAEs) due to global variations in spontaneous reporting practices. Accordingly, all such analyses were conducted exclusively using FAERS data.

In summary, the disease spectrum of osimertinib-related CAEs in the real world can be summarized as follows: cardiac function impairment-related diseases: cardiac failure, congestive cardiac failure, cardiac dysfunction, acute cardiac failure, and heart failure with reduced ejection fraction; arrhythmia-related diseases: atrial fibrillation and Long QT syndrome; myocardial disease-related diseases: toxic cardiomyopathy, stress cardiomyopathy, and cardiomyopathy; and other types of cardiac diseases: pericardial effusion, cardiotoxicity, and cardiac disorder. ​Of note, the disproportionality signals identified in FAERS and VigiBase analyses indicate potential statistical associations rather than confirming a direct causal or drug-induced toxicological mechanism. Spontaneous reporting data reflect observed reporting events rather than definitive evidence of causality, and these findings should be interpreted as preliminary clues that warrant further experimental validation.

### 3.3 Screening of targets for osimertinib-induced CAEs and PPI network analysis based on network pharmacology

Based on the above disease spectrum, a total of 2,924 related disease targets were retrieved through the GeneCard database; meanwhile, the chemical structure of osimertinib (SMILE = CN1C = C (C2 = CC = CC = C21) C3 = NC (=NC = C3) NC4 = C (C=C (C (=C4) NC (=O) C=C) N (C) CCN (C) C) OC) was obtained from the PubChem database, and target prediction was performed on three platforms, namely, SuperPredTargets, SwissTargetPrediction, and SEA, based on this structure, resulting in a total of 273 predicted targets. By considering the intersection of the predicted targets of osimertinib and disease-related targets, 111 intersection targets were obtained, which were used as research objects for subsequent network pharmacology (Supplementary Figure S1; Supplementary Table S1).

**FIGURE 5:**
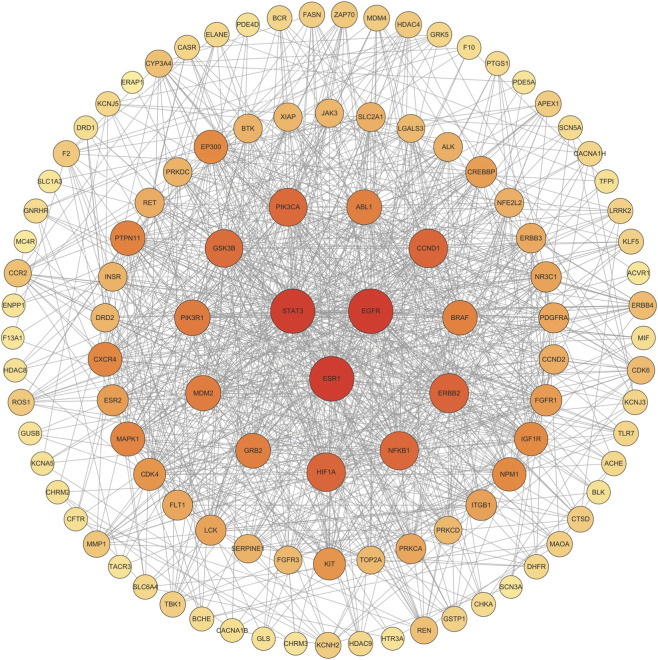
Protein-protein interaction network diagram of intersection targets between CAEs and Osimertinib.

Subsequently, we conducted a PPI analysis of the 111 intersection targets using the STRING database and drew a PPI diagram using Cytoscape software. Analysis of the network node connectivity revealed that EGFR, ESR1 (connectivity 66), and STAT3 (65) were core hubs, whereas ERBB2 (50), CCND1 (49), HIF1A (49), NFKB1 (48), and PIK3CA (47) were sub-core nodes (Figure 5).

**FIGURE 6:**
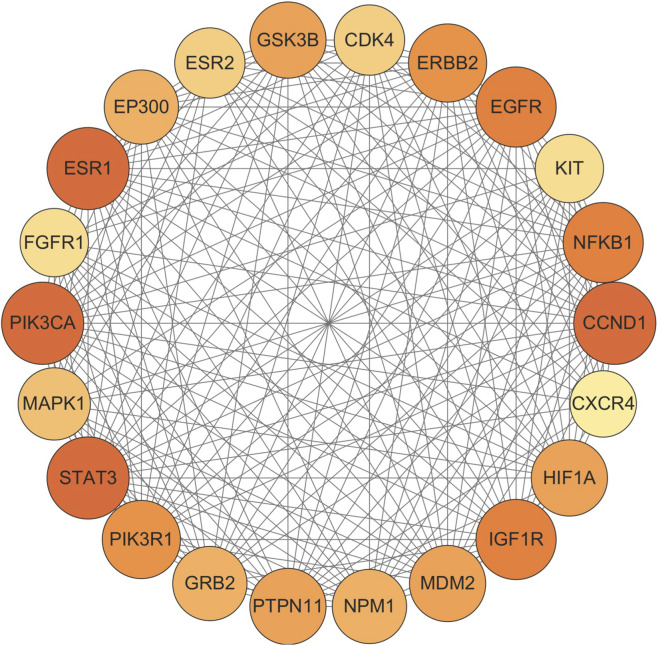
Protein-protein interaction network diagram of core targets.

Thereafter, with “Betweenness Centrality > Median; Closeness Centrality > Median; Degree >2 × Median” as the screening criteria for core targets, a total of 22 core targets were screened out. A PPI network of core targets was constructed and topological analysis was performed, which revealed that ESR1, STAT3, PIK3CA, and CCND1 formed a core hub with degree = 21 and cooperated with EGFR, IGF1R, NFKB1 (degree = 20), ERBB2, and PIK3R1 (degree = 19), along with GSK3B, MDM2, and HIF1A (Degree = 17–18) to synergistically mediate core signal interactions (Figure 6).

### 3.4 GO and KEGG enrichment analyses of intersection targets and core targets

**FIGURE 7:**
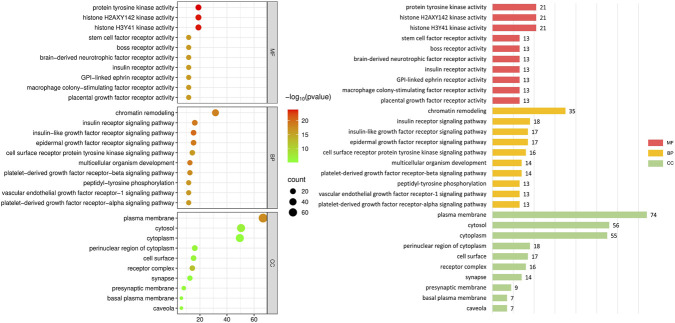
GO enrichment analysis for intersection targets (Top 10).

An enrichment analysis was performed on 111 target intersections. It should be noted that the above GO and KEGG enrichment results provide supportive biological evidence rather than definitive pathway-specific proof, as such enriched signaling events are commonly observed in broad-spectrum target enrichment analyses. GO enrichment analysis (Figure 7) showed that, in terms of molecular function, protein tyrosine kinase activity and histone kinase activity were the core, and they involved growth factor receptor activities, such as stem cell factor and brain-derived neurotrophic factor, suggesting that the targets play a role through kinase-catalyzed and receptor-mediated signal transduction. For biological processes, the focus was on chromatin remodeling and growth factor signaling pathways, such as insulin, epidermal growth factor, platelet-derived growth factor (PDGF), and multicellular organism development, reflecting the regulation of the targets on gene expression, cell proliferation, and differentiation. Regarding cellular components, the targets were mainly localized in the plasma membrane, cytosol, and cytoplasm and also involved receptor complexes in the perinuclear cytoplasm and cell surface, showing the distribution characteristics of membrane-bound or intracellular localization.

**FIGURE 8:**
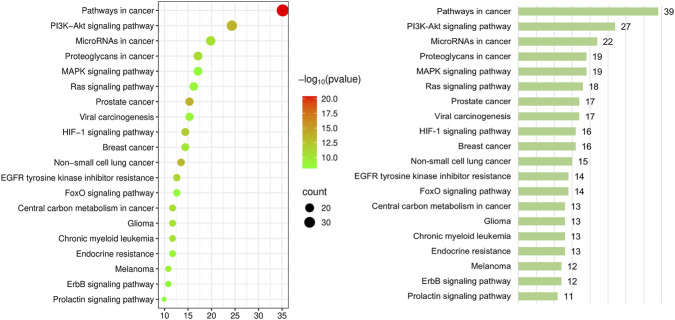
KEGG enrichment analysis for intersection targets (Top 20).

KEGG enrichment analysis (Figure 8) showed that KEGG enrichment was centered on cancer-related pathways; pathways in cancer were the most notably enriched (39 genes), and it also included tumor-specific pathways, such as prostate cancer and breast cancer. In addition, it involved EGFR-TKI resistance (14 genes) and central carbon metabolism in cancer (13 genes), suggesting that the targets are associated with osimertinib resistance and metabolic reprogramming. From the perspective of cardiac physiology, pathways such as PI3K-Akt and MAPK are involved in the maintenance of cardiac function. It has been speculated that osimertinib induces AEs by inhibiting these targets and interfering with cardiac signal/metabolic regulation.

**FIGURE 9:**
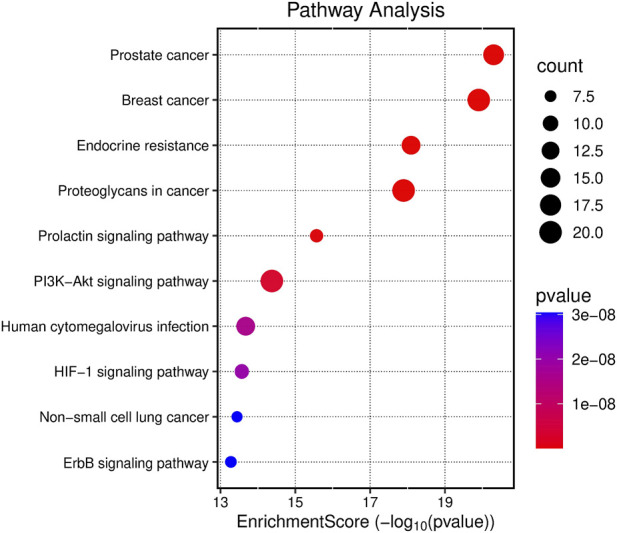
KEGG enrichment analysis for core targets (Top 10).

In addition, the KEGG enrichment analysis of the 22 core targets showed that the enrichment significance of prostate cancer and breast cancer pathways was the highest, which were the most core associated pathways of the targets. The enrichment degrees of the PI3K-Akt signaling pathway, endocrine resistance, and cancer-related proteoglycan pathway were next, reflecting that the targets were involved in carcinogenic signal transduction and drug resistance mechanisms. The enrichment of pathways, such as human cytomegalovirus infection, HIF-1 signaling, and ErbB signaling, was relatively weak. Pathways such as PI3K-Akt and ErbB simultaneously regulate myocardial survival and proliferation, suggesting that osimertinib may induce CAEs by inhibiting these targets and pathways (Figure 9).

### 3.5 Validation of targets via summary data-based mendelian randomization

Through retrieval and screening, five valid GWAS datasets were successfully retrieved from the FinnGen database: “HEARTFAIL” (corresponding to Heart failure), “AF” (corresponding to Atrial fibrillation and flutter), “CARDMYO” (corresponding to Cardiomyopathy), “MYOCARD” (corresponding to Myocarditis), and “PAROXTAC” (corresponding to Paroxysmal tachycardia). The eQTL data obtained from GTEx were GTEx_V8_cis_eqtl_summary_lite (hg19) [only Single Nucleotide Polymorphisms with P < 1e-5 were included].

**FIGURE 10:**
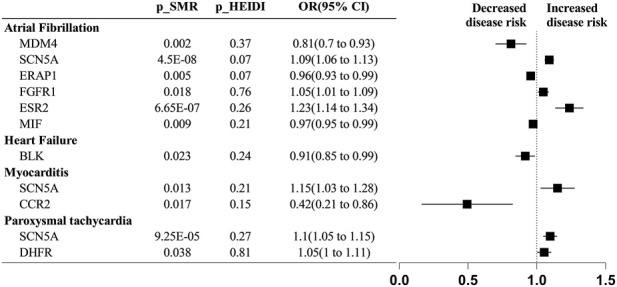
Forest plot for Mendelian randomization results for genes in relation to cardiovascular disease.

The results of the SMR analysis of 111 genes showed that in atrial fibrillation (Supplementary Figure S2), six genes, including MDM4, SCN5A, ERAP1, FGFR1, ESR2, and MIF, were notably associated (p_SMR<0.05). Among them, SCN5A (p_SMR = 4.5 × 10^−8^) and ESR2 (p_SMR = 6.65 × 10^−7^) were highly prominent, with OR values of 1.09 (1.06–1.13) and 1.23 (1.14–1.34), respectively, which were risk factors; MDM4, ERAP1, and MIF were protective factors (OR<1). In heart failure (Supplementary Figure S3), only BLK showed a meaningful association (p_SMR = 0.023) with OR = 0.91 (0.85–0.99), acting as a protective factor. In myocarditis (Supplementary Figure S4), SCN5A (p_SMR = 0.013, OR = 1.15) and CCR2 (p_SMR = 0.017, OR = 0.42) exhibited marked associations, functioning as a risk and strong protective factor, respectively. In paroxysmal tachycardia Supplementary Figure S5), SCN5A (p_SMR = 9.25 × 10^−5^, OR = 1.10) and DHFR (p_SMR = 0.038, OR = 1.05) were clearly associated, both being risk factors. The p_HEIDI values of all of the above genes were >0.05 [no notable pleiotropy] (Figure 10).

Although the nine targets verified by SMR were too few for an enrichment analysis, an in-depth review of previous studies revealed that most of these targets (including MDM4, SCN5A, ESR2, MIF, FGFR1, BLK, and CCR2) were directly related to the PI3K/AKT pathway—the same core mechanism axis we focused on in the animal experiment. Specifically, FGFR1 can activate the PI3K/AKT pathway through adapter protein mediation after activation by ligands (Liu et al., 2020); ESR2 inhibits this pathway by upregulating PTEN (Luo et al., 2023; Lindberg et al., 2011; Tian et al., 2020; Zhu et al., 2022); MIF promotes inflammation with PI3K/AKT as the downstream signal (Zhang et al., 2024); BLK, as an upstream molecule, is involved in the activation of the PI3K pathway (Lukas et al., 2020); the abnormal function of SCN5A is regulated by PI3K-α; and CCR2 affects inflammation and apoptosis by inhibiting this pathway (Tian et al., 2022). These targets interact with the PI3K/AKT pathway in multiple ways, such as activation, inhibition, and signal transduction, which is consistent with the regulatory role of the PI3K/AKT pathway in osimertinib-induced cardiotoxicity that we validated in the animal experiment. Notably, SMR analysis confirmed the genetic association of these genes with osimertinib-related cardiac adverse events (e.g., atrial fibrillation, myocarditis), while our animal experiment further verified that targeting the PI3K/AKT pathway (and its associated molecules) can regulate osimertinib-induced cardiac injury. This cross-validation between human genetic evidence (SMR) and *in vivo* experimental data (animal study) strongly supports that the PI3K/AKT pathway is a conserved, core mechanism axis underlying osimertinib-induced cardiotoxicity. Furthermore, the KEGG enrichment analysis of network pharmacology has suggested that the PI3K/AKT pathway is a key pathway associated with osimertinib-induced cardiotoxicity, and the results of the two—i.e., the findings from SMR target verification and the KEGG enrichment analysis—mutually confirmed each other. Therefore, we focused our research perspective on the PI3K/AKT pathway (Supplementary Figure S6), which is consistent with the mechanism exploration direction of our animal experiment.

Notably, KEGG pathway enrichment based on network pharmacology highlighted the PI3K/AKT pathway as one of the most significant pathways underlying osimertinib-induced cardiotoxicity. The consistent convergence of SMR-validated genes and network pharmacology findings supports the central role of the PI3K/AKT pathway. In addition, combined with our team’s previous work and established evidence in cardio-oncology, myeloperoxidase (MPO) has been functionally linked to the AMPK/PI3K/AKT/eNOS signaling network (Lukas et al., 2020; Tian et al., 2022; Li et al., 2022; Ma et al., 2024). This axis plays a critical role in regulating oxidative stress, inflammatory activation, and endothelial homeostasis, all of which are key pathological features of cardiotoxicity associated with targeted anticancer agents.

Based on this, combined with previous research by our team and known results in the field of antitumor drug toxicity, MPO is potentially associated with the AMPK/PI3K/AKT/eNOS pathway (18–21). This pathway network plays an important role in regulating oxidative stress, inflammatory response, and vascular endothelial function and is closely related to the cardiotoxicity mechanism of antitumor drugs. Therefore, subsequent research will further explore the interaction between osimertinib-induced cardiotoxicity and the AMPK/PI3K/AKT/eNOS pathway, along with MPO, to more systematically analyze its toxic molecular mechanism.

### 3.6 Effects of osimertinib on cardiac MPO expression and AMPK/PI3K/eNOS pathway in SD rats

**FIGURE 11:**
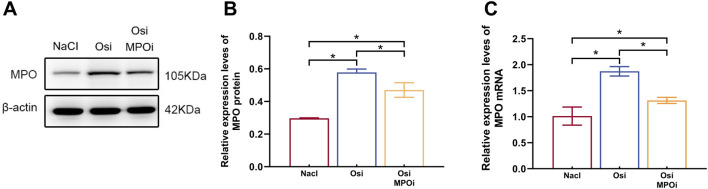
MPO protein and mRNA expressions with quantitative analysis in myocardial tissue of SD rats of different treatment groups. **(A)**: Western blot band of MPO protein expression; **(B)**: Quantitative histogram of MPO protein expression; **(C)**: Quantitative histogram of MPO mRNA expression.

To explore the mechanism of osimertinib (Osi)-induced CAEs, we established three treatment groups: normal saline (NaCl, control), osimertinib (Osi), and osimertinib combined with an MPO inhibitor (Osi + MPOi). The mRNA (qRT-PCR) and protein (Western Blot) expression levels of MPO were detected. The results showed that compared with the NaCl group, the protein (Figures 11A,B) and mRNA (Figure 11C) of MPO in the Osi group were notably increased (P < 0.05), whereas in the Osi + MPOi group, the mRNA and protein expression levels of MPO were markedly decreased compared with the Osi group (P < 0.05).

**FIGURE 12:**
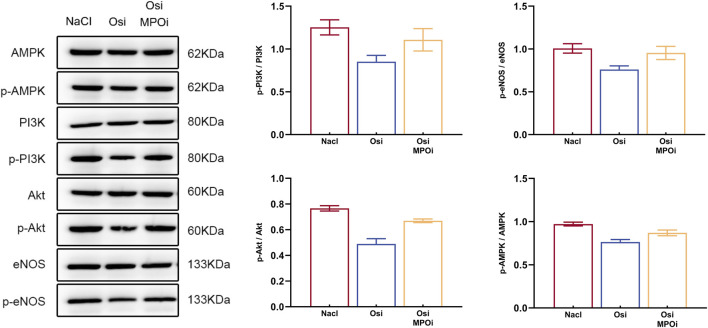
Western blot analysis of AMPK, PI3K, Akt, and eNOS and their phosphorylation in myocardial tissue of SD rats of different treatment groups.

To further explore the association between MPO and the AMPK/PI3K/Akt/eNOS signaling pathway during osimertinib-induced cardiac injury, the phosphorylation levels of key molecules in this pathway (quantified by the ratio of phosphorylated protein to total protein) were measured. Western blot and grayscale analysis showed that compared with the NaCl control group, the relative phosphorylation levels of p-AMPK, p-PI3K, p-Akt, and p-eNOS in the osimertinib (Osi)-treated group were notably decreased (P < 0.05), suggesting that osimertinib can inhibit the phosphorylation activation of this pathway; after intervention with osimertinib combined with MPO inhibitor (Osi + MPOi group), the relative levels of the above phosphorylated proteins were markedly increased compared with the Osi group (P < 0.05), indicating that MPO inhibitor can partially antagonize the inhibitory effect of osimertinib on the AMPK/PI3K/Akt/eNOS pathway (Figure 12).

Combined with the previous finding that MPO expression was upregulated in rat cardiac tissue after osimertinib treatment, it is speculated that osimertinib can inhibit the activation of the AMPK/PI3K/Akt/eNOS pathway by upregulating MPO.

### 3.7 MPO inhibition alleviates osimertinib-induced cardiac electrophysiological and structural-functional damage, whereas the inhibition of AMPK/PI3K/eNOS pathway counteracts its protective effect

**FIGURE 13:**
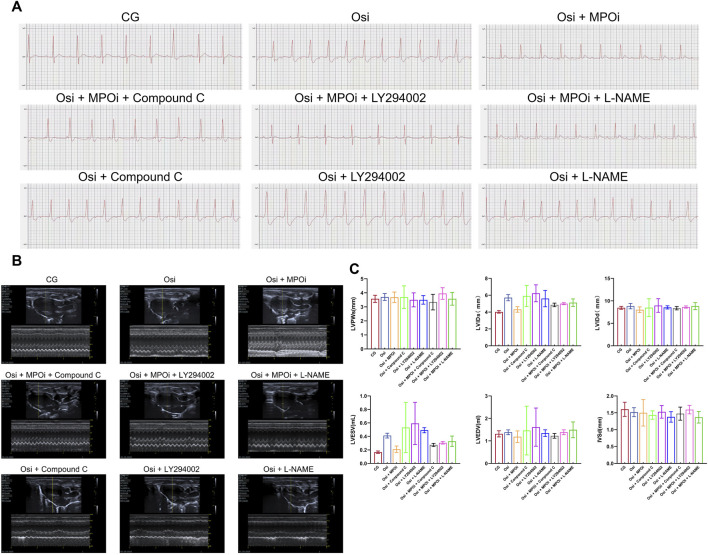
ECG and echocardiographic results of SD rats in various treatment groups. **(A)**: Electrocardiogram images of each group; **(B)**: Echocardiographic images of each group; **(C)**: Histograms of cardiac ultrasound related indicators in each group.

The ECG results (Figure 13A) showed revealed the following: the control group had regular heart rhythm, and the morphology of QRS complex, ST segment, and T wave conformed to physiological characteristics, with good maintenance of myocardial electrical activity homeostasis; the Osi-treated group had accelerated heart rate, widened QRS complex, and ST segment deviation accompanied by abnormal T wave morphology, suggesting myocardial electrophysiological disorders. After treatment with osimertinib combined with an MPO inhibitor (Osi + MPOi), the regularity of heart rhythm improved, the QRS complex and ST-T waveforms approached those of the control group (CG), and electrophysiological abnormalities were significantly alleviated. After further introduction of an AMPK inhibitor (Compound C), PI3K inhibitor (LY294002) or eNOS inhibitor (L-NAME), arrhythmia and waveform distortion reappeared in the electrocardiographic phenotype of the Osi + MPOi group, and the damage characteristics were similar to those of the Osi group. The electrocardiographic phenotype of the group directly treated with the Osi + pathway inhibitor did not eliminate the disordered pattern.

The quantitative results of echocardiography (Figure 13B) showed that: the LVEF and LVFS in the CG group maintained normal levels, the stroke volume (SV) was stable (Figure 13C), and the left ventricular end-diastolic volume (LVEDV), end-systolic volume (LVESV), interventricular septal end-diastolic thickness (IVSd), left ventricular end-diastolic diameter (LVIDd), end-systolic diameter (LVIDs), and posterior wall end-diastolic thickness (LVPWs) all conformed to the physiological baseline (Figure 13D). In the Osi-treated group, LVEF and LVFS were notably decreased (P < 0.05); SV was decreased; LVEDV and LVESV were increased (ventricular dilation); IVSd, LVIDd, and LVIDs were disordered; and abnormal LVPWs suggested myocardial structural damage. After Osi + MPOi treatment, the systolic function, volume, and structural indices all approached the baseline of the CG group, and cardiac structural and functional damage was markedly repaired. However, when Osi + MPOi was combined with pathway inhibitors (Compound C/LY294002/L-NAME) for intervention, LVEF and LVFS decreased again, and the volume and structural indexes showed dilation and deformity characteristics; the group directly treated with Osi + pathway inhibitor also showed similar damage phenotypes.

### 3.8 MPO inhibition alleviates osimertinib-induced myocardial structural damage, apoptosis, and membrane injury, whereas inhibition of AMPK/PI3K/eNOS pathway counteracts its protective effect

The results of H&E staining and TUNEL apoptosis rate quantification showed that in the control group (CG), myocardial fibers were arranged regularly, cell morphology was normal, and the apoptosis rate was maintained at the baseline level. In the osimertinib (Osi) group, myocardial fibers were disordered, cells were swollen and deformed, and the apoptosis rate was notably increased (P < 0.05), suggesting that myocardial structural damage and cell apoptosis occurred synergistically. In the osimertinib combined with MPO inhibitor (Osi + MPOi) group, the arrangement of myocardial fibers and cell morphology were close to those of the CG group, and the apoptosis rate was markedly decreased (P < 0.05), showing synchronous repair of structural and apoptotic damage; after treatment with Osi + MPOi combined with AMPK inhibitor (Compound C), PI3K inhibitor (LY294002) or eNOS inhibitor (L-NAME), or directly with Osi + the above pathway inhibitors, myocardial fibers were re-disordered, cell morphology was re-deformed again, and the apoptosis rate rose to the level of the Osi group (P < 0.05), with the damage phenotype recurring (Supplementary Figure S7).

**FIGURE 14:**
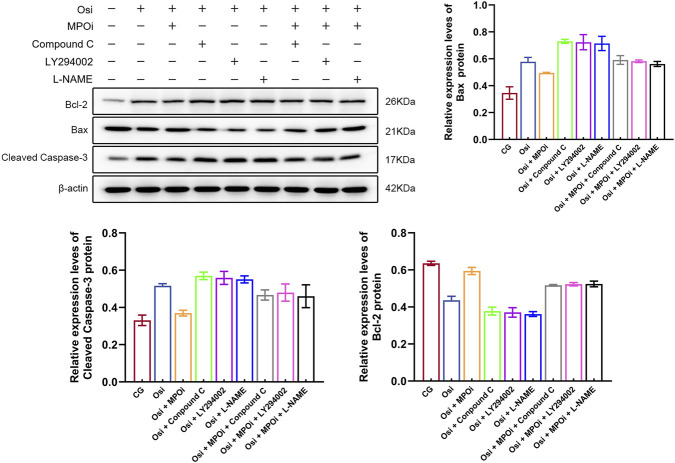
Western blot analysis of apoptosis-related proteins in myocardial tissue of SD rats across different treatment groups.

Western blot and quantitative analysis of apoptosis-related proteins showed that in the CG, the anti-apoptotic protein Bcl-2 was highly expressed, whereas the pro-apoptotic protein Bax and the apoptosis executive molecule Cleaved Caspase-3 were lowly expressed; in the Osi group, Bcl-2 was notably decreased (P < 0.05) and Bax and Cleaved Caspase-3 were markedly increased (P < 0.05); in the Osi + MPOi group, Bcl-2 was increased (P < 0.05) and Bax and Cleaved Caspase-3 were decreased (P < 0.05). After intervention with these pathway inhibitors, Bcl-2 decreased again and Bax and Cleaved Caspase-3 were highly expressed again, with the change trend approaching that of the Osi group (P < 0.05) (Figure 14).

Quantitative analysis of lactate dehydrogenase (LDH) and creatine kinase (CK) showed that in the CG, LDH and CK maintained low baseline values, indicating good myocardial cell integrity; in the Osi group, both were notably increased (P < 0.05), indicating myocardial cell damage and increased membrane permeability; in the Osi + MPOi group, LDH and CK were markedly decreased (P < 0.05), indicating alleviated myocardial membrane damage. After intervention with the above pathway inhibitors, LDH and CK increased again, with the change trend approaching that of the Osi group [P < 0.05] (Supplementary Figure S8).

### 3.9 MPO inhibition alleviates osimertinib-induced myocardial oxidative stress and inflammatory response, whereas inhibition of AMPK/PI3K/eNOS pathway counteracts its protective effect

The detection results of oxidative stress indicators (MDA, ROS, SOD, GSH-PX) showed that the control group had low levels of MDA and ROS and high activities of SOD and GSH-PX; the Osi group had notably increased MDA and ROS (P < 0.05) and markedly decreased activities of SOD and GSH-PX (P < 0.05); the Osi + MPOi group had notably decreased MDA and ROS (P < 0.05) and markedly increased activities of SOD and GSH-PX (P < 0.05). After treatment with Osi + MPOi combined with AMPK/PI3K/eNOS pathway inhibitors (Compound C/LY294002/L-NAME) or directly with Osi + the above pathway inhibitors, MDA and ROS increased again and the activities of SOD and GSH-PX decreased, with the change trend approaching that of the Osi group [P < 0.05] (Supplementary Figure S9).

The detection results of proinflammatory factors (IL-1β, IL-6, TNF-α) showed that the control group had low levels of IL-1β, IL-6, and TNF-α; the Osi group had notably increased levels of the three (P < 0.05); the Osi + MPOi group had markedly decreased levels of IL-1β, IL-6, and TNF-α (P < 0.05). After treatment with Osi + MPOi combined with the above pathway inhibitors or directly with Osi + the above pathway inhibitors, IL-1β, IL-6, and TNF-α increased again, with the change trend approaching that of the Osi group (P < 0.05) (Supplementary Figure S10).

## 4 Discussion

**FIGURE 15:**
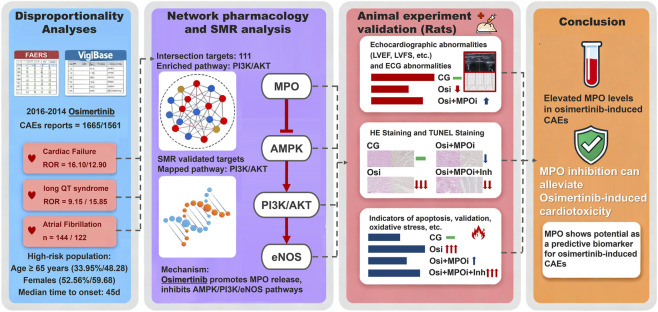
Integrated workflow diagram of this study.

Through multidimensional verification including real-world clinical database analysis, network pharmacology, Mendelian randomization, and animal experiments, this study systematically revealed the clinical characteristics and molecular mechanisms of Osimertinib-related CAEs, thereby providing key evidence for clinically safe medication and intervention strategies (Figure 15).

### 4.1 Clinical characteristics and risk factors of osimertinib-related CAEs

Real-world data (FAERS and VigiBase) clearly indicate that the disease spectrum of Osimertinib-related CV-AEs mainly includes cardiac dysfunction, arrhythmias (such as atrial fibrillation and long QT syndrome), and myocardial diseases. Heart failure with reduced ejection fraction (ROR = 47.76) and cardiac dysfunction (ROR = 16.10) showed high-intensity safety signals, suggesting a close association with drug exposure. Population characteristic analysis showed that older patients over 65 years of age (accounting for 48.28%) and females (accounting for 52.56%–59.68%) were high-risk groups, and the hospitalization (48.2%) and mortality rates (11.11%–45.45%) of CV-AEs were markedly higher than those of overall AEs. Time-trend analysis revealed that the median onset time of CV-AEs was 45 days, which was shorter than 59 days of overall AEs, suggesting that cardiac damage can occur in the early stage of treatment, supporting the clinical practice of strengthening cardiac function assessment within 1–2 months after medication.

### 4.2 Core position of PI3K/AKT pathway and target regulatory network

Through the cross-validation of network pharmacology and Mendelian randomization, we provide integrated, multi-layered suggestive evidence that the PI3K/AKT pathway may serve as a key signaling axis potentially linked to osimertinib-induced cardiotoxicity. Among the 111 intersection targets, 22 core targets including SCN5A and PIK3CA constituted a closely connected module in the PPI network, the majority of which were enriched in the PI3K/AKT signaling cascade. As a potential limitation of this study, ESR1 was identified as a highly connected hub node through unbiased *in silico* screening; however, its biological relevance may be limited in the male SD rat model used in this study, given the relatively weak role of estrogen-related signaling in the male cardiac environment. Since the present study focused on the overall trend and functional enrichment of the PI3K/AKT pathway rather than individual sex-specific targets, the potential influence of this sex-related bias on the overall interpretation was reduced. Among these key targets, SCN5A was regarded as the more biologically plausible hub gene in male animals, which may participate in maintaining myocardial electrophysiological homeostasis possibly by modulating the PI3K/AKT pathway. Mendelian randomization further suggested that nine targets, including SCN5A and ESR2, may be associated with the PI3K/AKT pathway (e.g., ESR2 may suppress pathway activity by upregulating PTEN), supporting the potential genetic relevance of this pathway.

It should be acknowledged that the present study is based on *in silico* prediction, observational enrichment analysis, and genetic correlation inference, which can indicate potential associations and generate mechanistic hypotheses but do not constitute definitive causal evidence. Despite this inherent limitation, our findings offer novel and experimentally testable insights into the potential mechanisms underlying osimertinib-induced cardiotoxicity. The PI3K/AKT pathway has been widely implicated in myocardial cell proliferation, anti-apoptosis, and vascular endothelial function (Li et al., 2022; Ma et al., 2024), and inhibition of this pathway by osimertinib may represent a biologically plausible mechanism that disrupts cardiac homeostasis, providing a novel, systematic, and genetically informed perspective and important directional clues for future experimental and clinical research.

### 4.3 Innovation and verification of the “MPO→AMPK/PI3K/enos pathway” cascade mechanism

Subsequent animal experiments first revealed the cascade mechanism of “Osimertinib→MPO upregulation→AMPK/PI3K/eNOS pathway inhibition,” providing a complete evidence chain from upstream regulation to downstream effects for osimertinib-induced cardiotoxicity. After osimertinib treatment, the expression of myocardial MPO in SD rats was notably increased, accompanied by a marked decrease in the phosphorylation levels of the AMPK/PI3K/eNOS pathway (p-AMPK, p-PI3K, etc.). MPO inhibitors reversed this effect and ameliorated cardiac electrophysiological disorders, structural damage, and functional decline. Further studies confirmed that co-administration with AMPK/PI3K/eNOS pathway inhibitors largely abolished the protective effect of MPO inhibitors. These findings indicate that the AMPK/PI3K/Akt/eNOS pathway may act as a key downstream signaling module mediating MPO-related cardiotoxicity. Although we cannot fully exclude the possibility that these pathways represent parallel stress-responsive pathways simultaneously activated by osimertinib exposure, the complete abrogation of protection by pathway inhibition supports a functional hierarchy in which MPO functions upstream of the AMPK/PI3K/Akt/eNOS axis. Further mechanistic investigations will be required to validate the precise regulatory relationship between these molecules.

The novelty of this mechanism is that MPO, as a key regulator of oxidative stress and inflammation, has mostly been focused on its role in anthracycline-induced cardiomyopathy in previous studies (Fu et al., 2022; Nettersheim et al., 2023; Milano et al., 2020; Etwebi et al., 2018), whereas this study is the first to link it with osimertinib-induced cardiotoxicity, expanding the role of MPO in targeted drug toxicity. The synergistic regulation of the AMPK/PI3K/eNOS pathway illustrates how osimertinib may affect the heart through MPO signaling, providing a potential theoretical basis for multi-target intervention strategies.

The results of this study provide specific guidance for risk management and intervention strategies in the clinical application of osimertinib. Regarding risk monitoring, it is recommended that for high-risk groups such as patients aged 65 years and older and female patients, close monitoring of high-risk events such as long QT syndrome and heart failure using electrocardiography (focusing on QT interval) and echocardiography (paying attention to LVEF, LVFS, etc.) within 45 days after administration is warranted. MPO level may serve as a potential biomarker for early warning of cardiotoxicity. Regarding therapeutic exploration, MPO inhibitors show promise in alleviating osimertinib-related cardiotoxicity, especially in populations with high MPO expression, offering a potential candidate strategy to reduce treatment discontinuation and ensure the continuity of antitumor therapy.

### 4.4 Limitations

This study has certain limitations. As FAERS and VigiBase are spontaneous reporting systems, they have inherent limitations in evaluating the true incidence of adverse events, and are susceptible to reporting bias, underreporting, confounding by concomitant medications, and incomplete clinical information. In addition, key clinical data such as exact dosage and comorbidities were incomplete, which may affect the precise estimation of the association between osimertinib and cardiovascular adverse events. Although information on concomitant medications was available, their limited completeness and granularity precluded robust multivariable adjustment; thus, the potential confounding effects of other cardiotoxic agents, including chemotherapies and cardiovascular drugs, were not fully accounted for.

For our *in vivo* experiments, only male SD rats were used to avoid confounding introduced by the estrous cycle and associated hormonal fluctuations in female animals, which could destabilize cardiac electrophysiological and toxicity measurements. This design enabled us to reduce experimental heterogeneity and improve the reliability and reproducibility of cardiotoxicity assessments. Nevertheless, cardiovascular phenotypes and drug responses may differ between sexes. Future studies including both male and female animals would help clarify potential sex-dependent differences and further enhance the translational relevance of our findings.

## Data Availability

The raw data supporting the conclusions of this article will be made available by the authors, without undue reservation.

## Supplementary material

SUPPLEMENTARY FIGURE S1 Venn diagram of overlap targets between cardiac AEs and Osimertinib.

SUPPLEMENTARY FIGURE S2 Mendelian randomization analysis of atrial fibrillation and gene expression levels.

SUPPLEMENTARY FIGURE S3 Mendelian randomization analysis of heart failure and gene expression levels.

SUPPLEMENTARY FIGURE S4 Mendelian randomization analysis of myocarditis and gene expression levels.

SUPPLEMENTARY FIGURE S5 Mendelian randomization analysis of paroxysmal tachycardia and gene expression levels.

SUPPLEMENTARY FIGURE S6 Gene mapping diagram of genes identified via Mendelian randomization in relation to the PI3KAKT pathway.

SUPPLEMENTARY FIGURE S7 H&E and TUNEL staining and quantitative apoptosis analysis in SD rat myocardium across groups.

SUPPLEMENTARY FIGURE S8 Quantitative analysis of myocardial injury markers in SD rats across treatment groups.

SUPPLEMENTARY FIGURE S9 Quantitative analysis of oxidative stress indicators across treatment groups.

SUPPLEMENTARY FIGURE S10 Quantitative analysis of proinflammatory cytokine in SD rats across treatment groups.
