# Supplementary figures and images for: Roles of myeloperoxidase and the AMPK/PI3K/AKT/eNOS pathway in osimertinib-induced cardiotoxicity: multilevel evidence from disequilibrium analysis, network pharmacology, mendelian randomization, and animal experiments
Source: Front Pharmacol. 2026 Jul 1;17:1776465. doi: 10.3389/fphar.2026.1776465 (PMC13368672; doi:10.3389/fphar.2026.1776465)

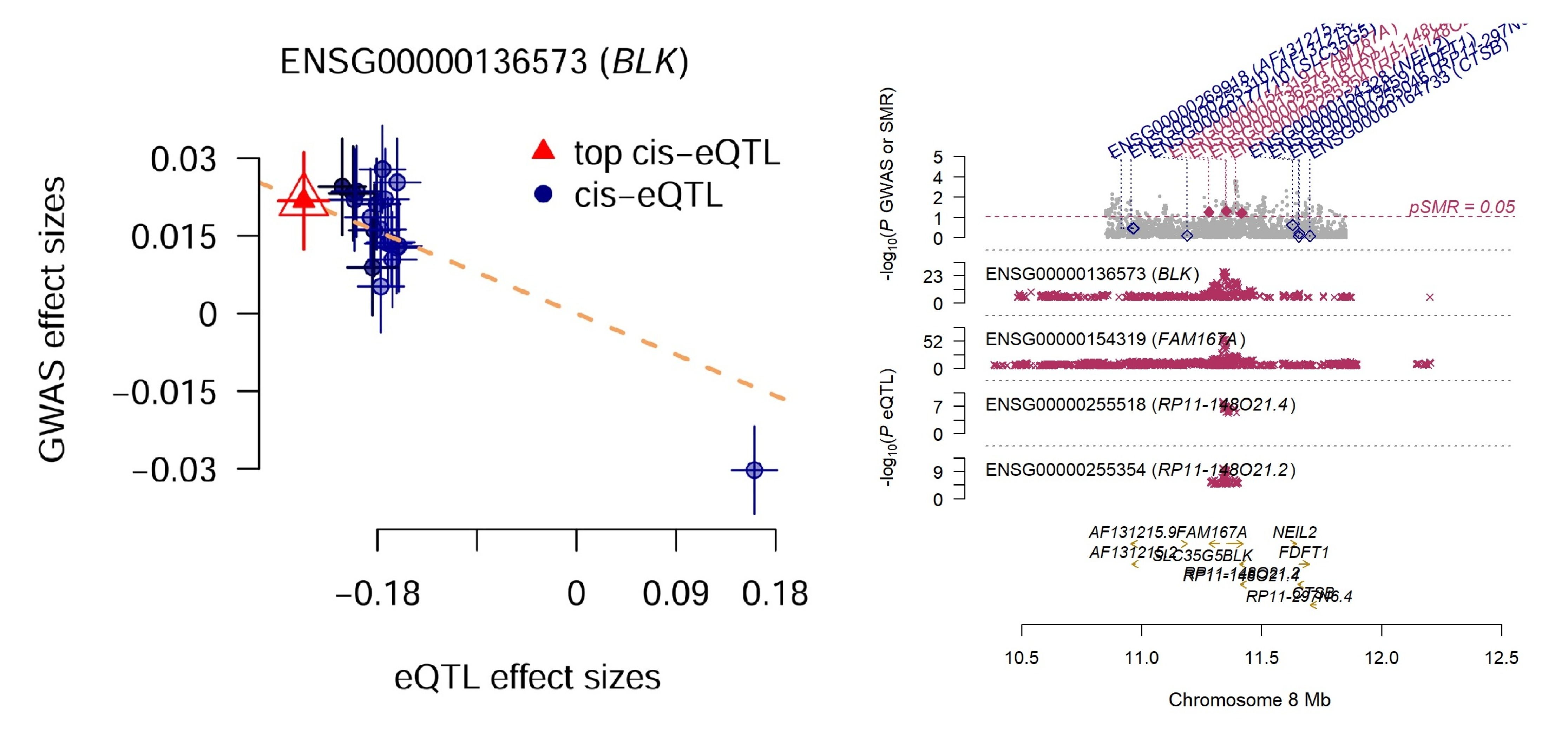

Supplement: Supplementary file 1 [file Image3.jpeg]

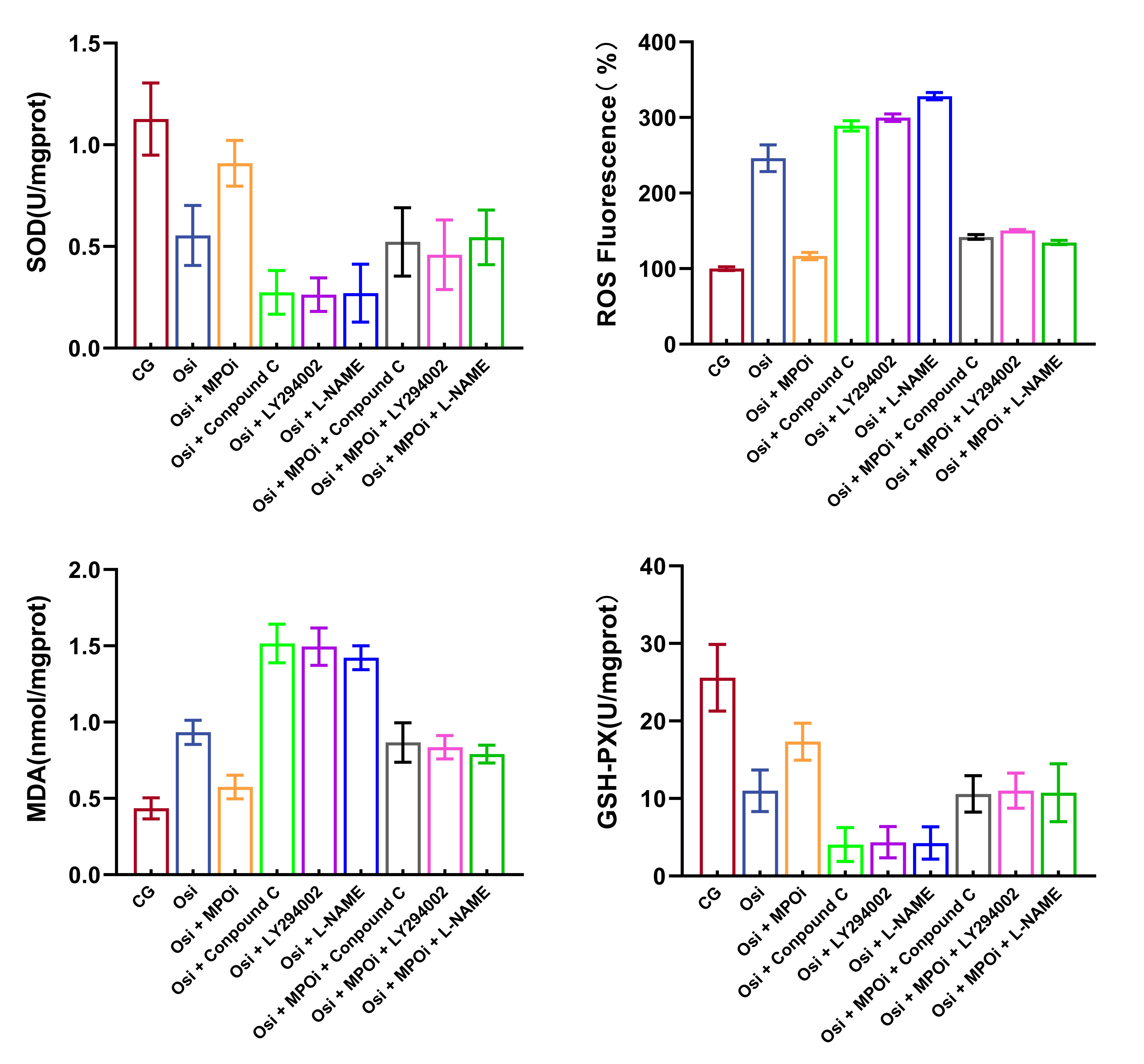

Supplement: Supplementary file 2 [file Image9.jpeg]

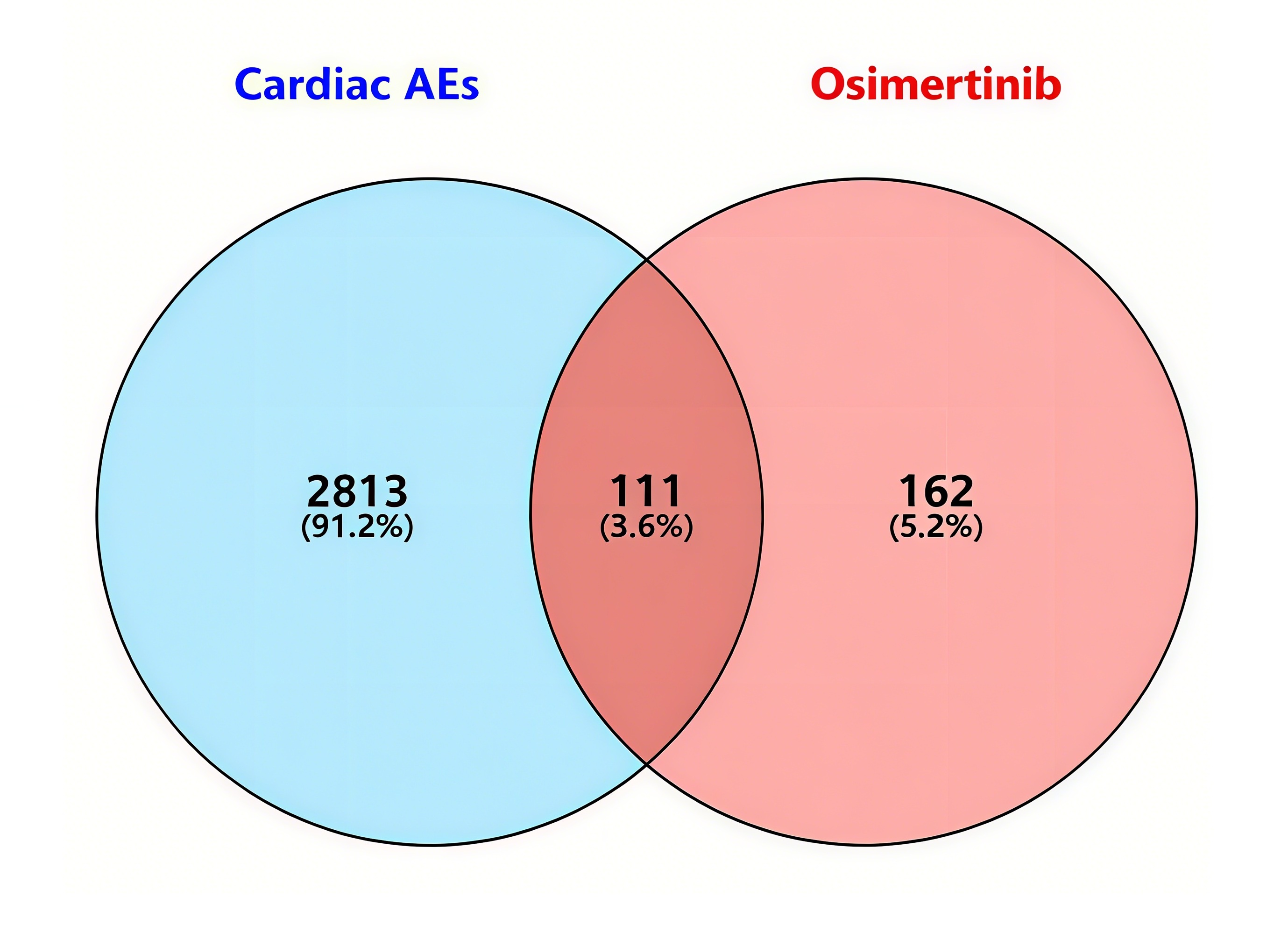

Supplement: Supplementary file 3 [file Image1.jpeg]

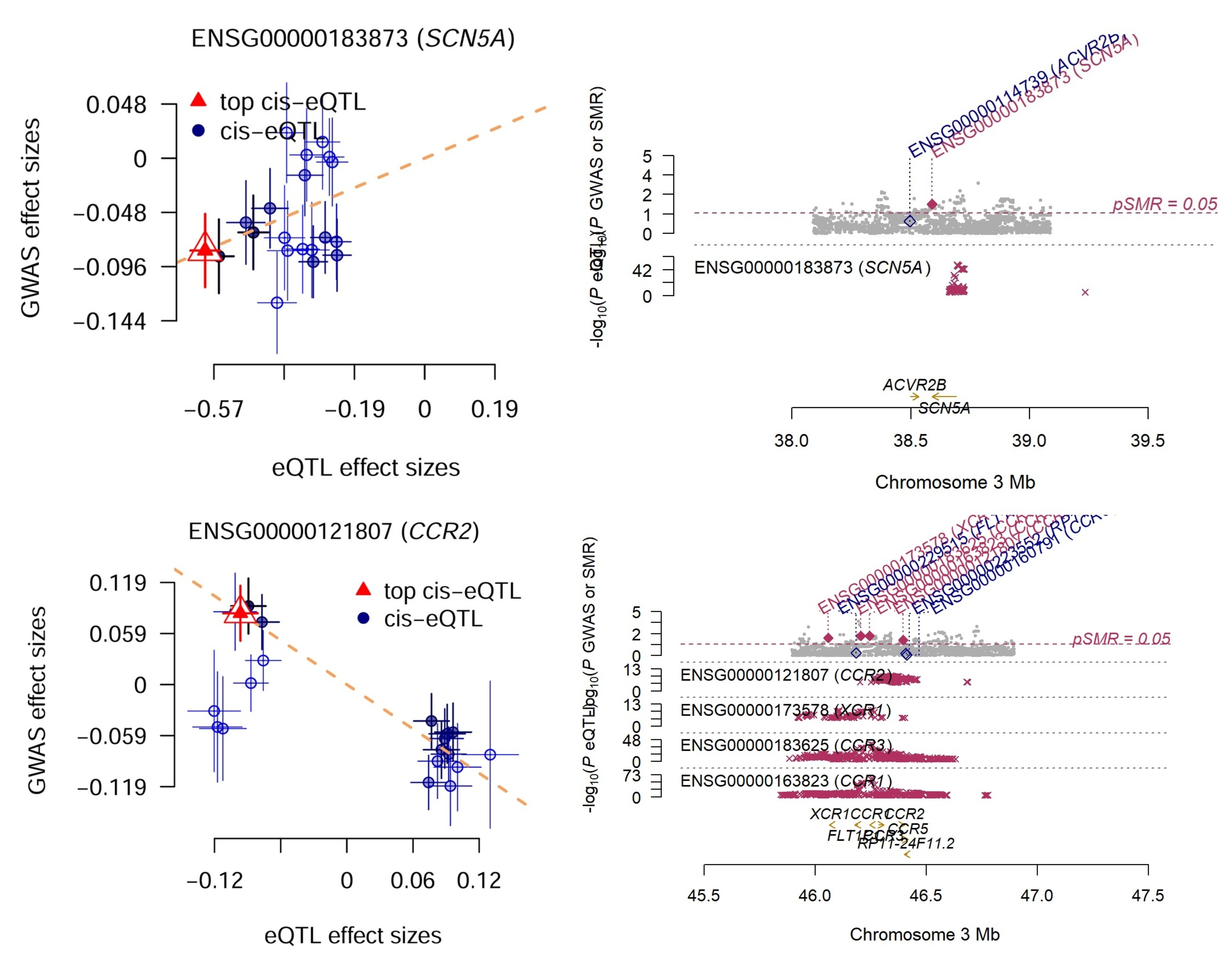

Supplement: Supplementary file 4 [file Image4.jpeg]

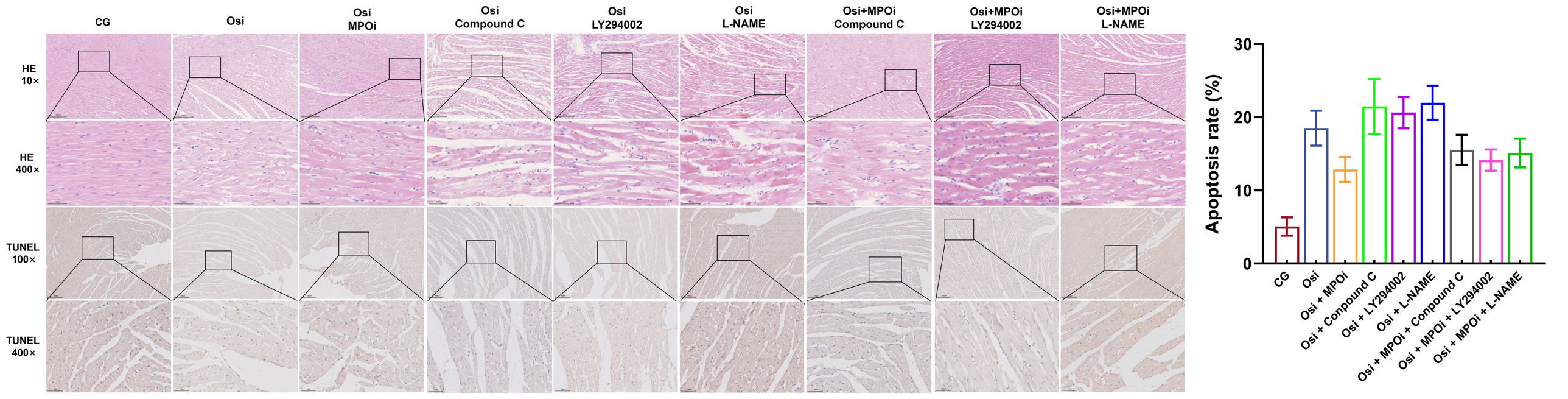

Supplement: Supplementary file 5 [file Image7.jpeg]

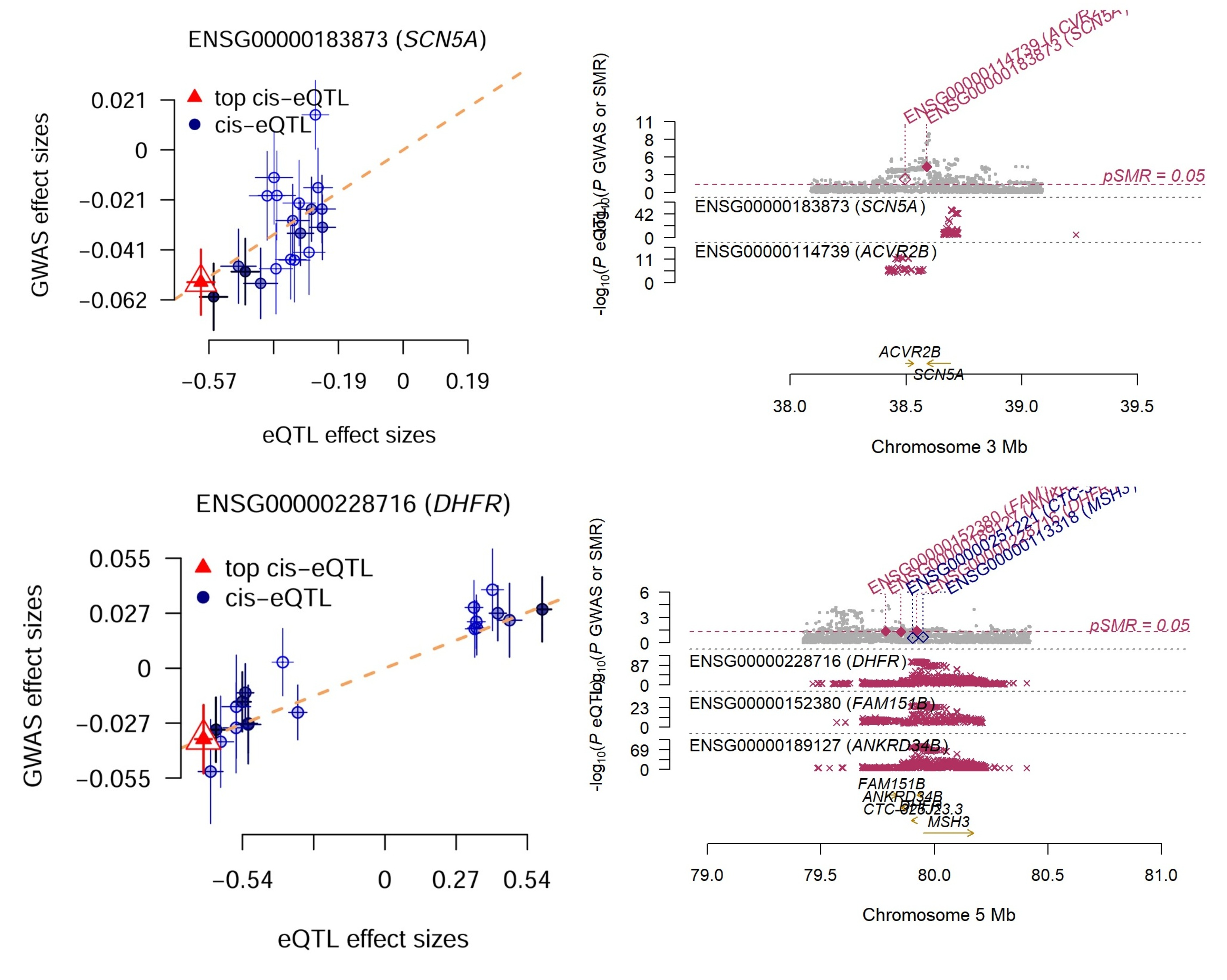

Supplement: Supplementary file 7 [file Image5.jpeg]

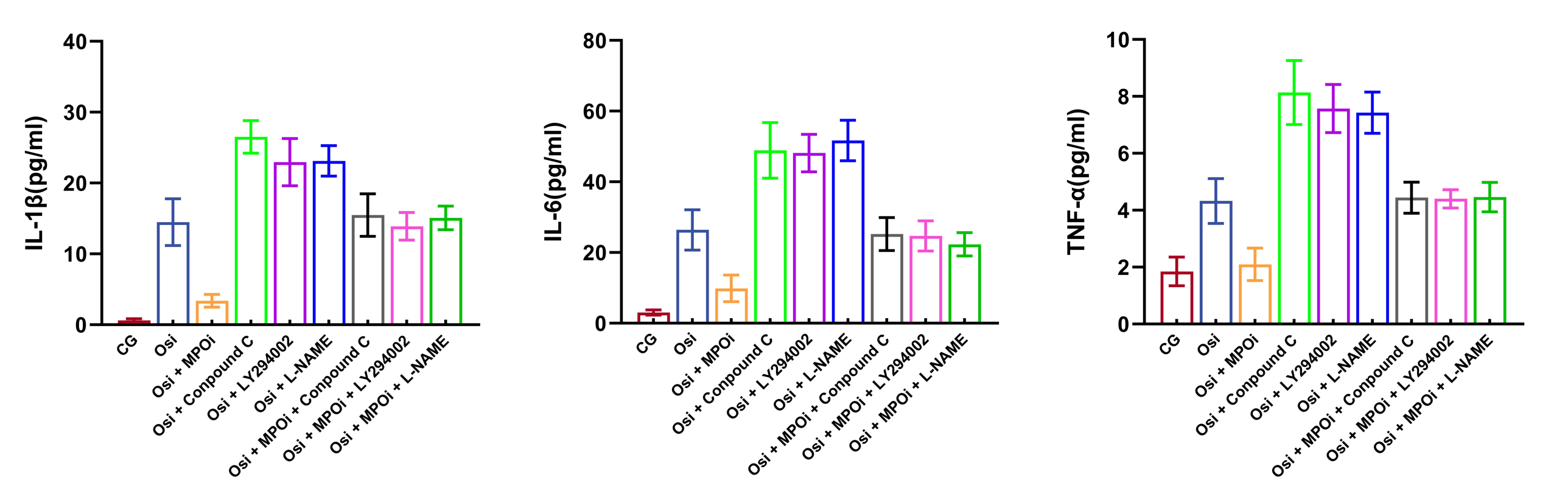

Supplement: Supplementary file 8 [file Image10.jpeg]

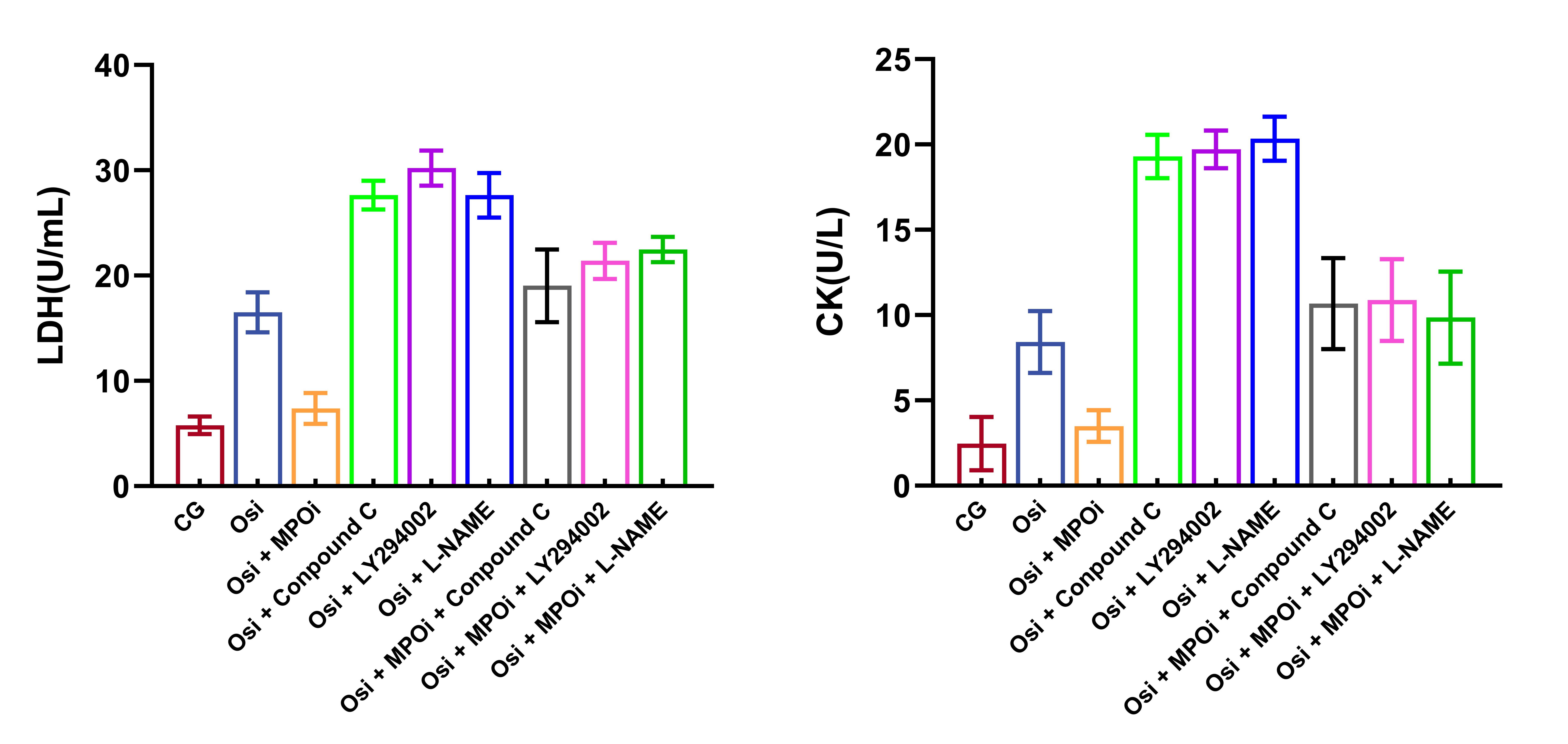

Supplement: Supplementary file 10 [file Image8.jpeg]

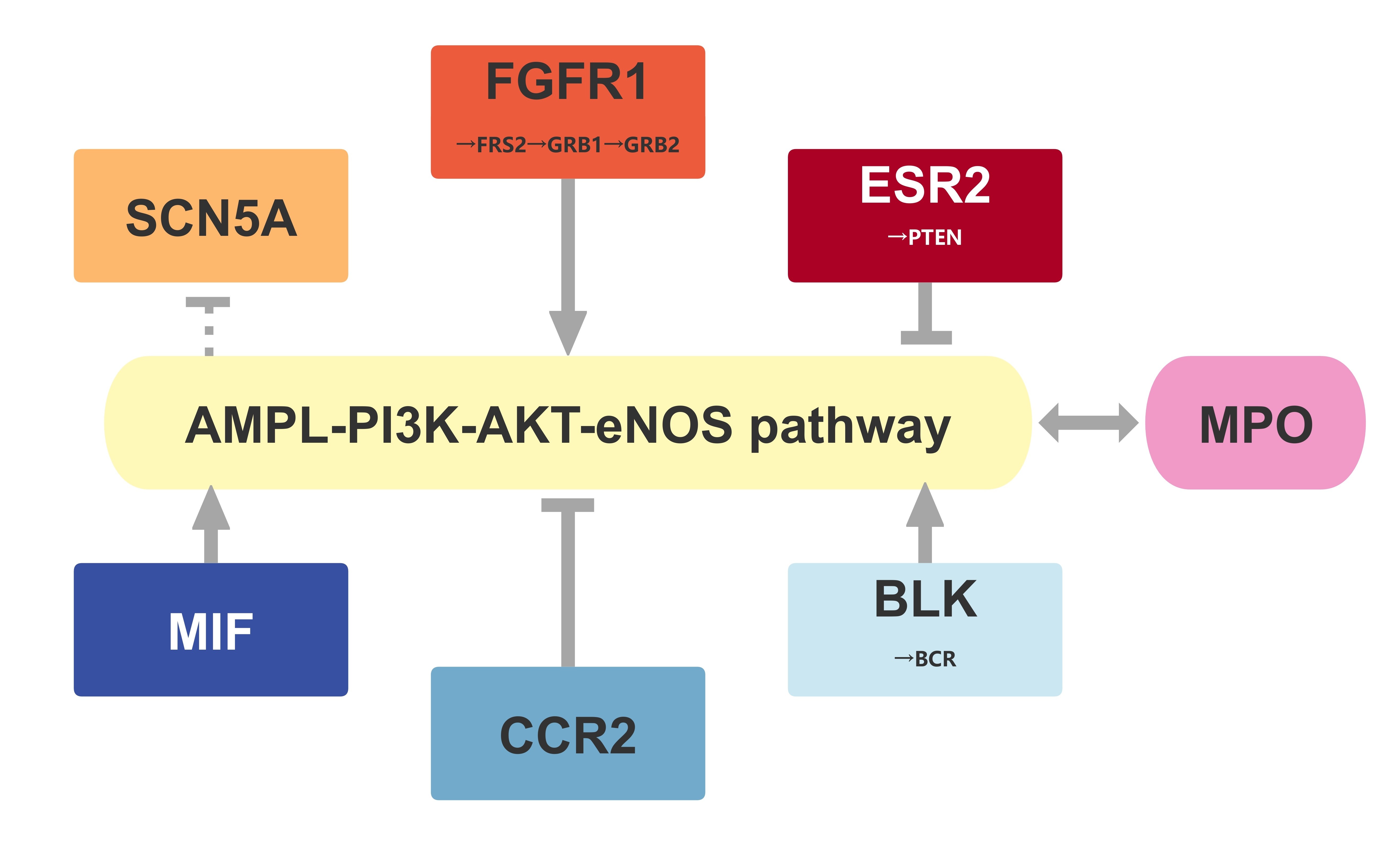

Supplement: Supplementary file 11 [file Image6.jpeg]
